# Association of drinking pattern with risk of coronary heart disease incidence in the middle-aged and older Chinese men: Results from the Dongfeng-Tongji cohort

**DOI:** 10.1371/journal.pone.0178070

**Published:** 2017-05-25

**Authors:** Yizhi Zhang, Yanqiu Yu, Yu Yuan, Kuai Yu, Handong Yang, Xiulou Li, Xinwen Min, Ce Zhang, Meian He, Xiaomin Zhang, Tangchun Wu

**Affiliations:** 1 Department of Occupational and Environmental Health, School of Public Health, Tongji Medical College, Huazhong University of Science & Technology, Wuhan, China; 2 Ministry of Education Key Lab for Environment and Health, School of Public Health, Tongji Medical College, Huazhong University of Science & Technology, Wuhan, China; 3 Department of Cardiovascular Diseases, Dongfeng Central Hospital, Hubei University of Medicine, Shiyan, China; Shanghai Diabetes Institute, CHINA

## Abstract

**Background:**

Epidemiologic studies have found that moderate alcohol consumption was associated with a decreased risk of coronary heart disease (CHD) incidence. Nevertheless, whether the drinking pattern is associated with CHD incidence still remains inconclusive.

**Methods:**

We included 8,469 Chinese men aged 45–81 years, who were free of CHD, stroke, or cancer at baseline from Dongfeng-Tongji cohort. A semi-structured questionnaire was used to collect information on alcohol consumption and other covariates. Cox proportional hazard regression model was applied to estimate the multivariable-adjusted hazard rations (HRs) and 95% confidence intervals (95% CIs).

**Results:**

During an average of 4.36 years of follow-up, we identified 959 incident CHD events. Compared with non-drinkers, the multivariable-adjusted HR (95% CI) of CHD incidence was 0.84 (0.71–0.98) in current drinkers. With respect to drinking pattern, men who consumed 20.01–40 grams ethanol once a time had a 24% lower risk of incident CHD (HR = 0.76, 95% CI = 0.62, 0.94) compared with non-drinkers. The adjusted HRs (95% CI) of CHD incidence were 0.80 (0.65, 0.99), 1.02 (0.84, 1.22), and 0.75 (0.59–0.96) in subjects who consumed 0.01–10, 10.01–30, and > 30 grams ethanol per day, respectively. Participants who consumed 20.01–40 grams ethanol per time with less than 5 times per week had the lowest risk of CHD incidence (HR = 0.73, 95% CI = 0.52, 0.96). No significant associations were observed between type or frequency of alcohol consumption and CHD incidence.

**Conclusions:**

Drinking was associated with a lower risk of CHD incidence in middle-aged and older Chinese men and moderate quantity of ethanol amounts once a time with lower frequency could been considered as a healthy drinking pattern, which might modify the relationship between alcohol consumption and incident CHD.

## Introduction

Coronary heart disease (CHD) has become the leading cause of deaths worldwide and global deaths from cardiovascular diseases have increased by 41% between the years 1990 and 2013 [1–3]. In China, the prevalence of CHD is also increasing rapidly and it has been expected that mortality from cardiovascular diseases would increase by 110% in men and 81% in women between 1990 and 2020 [4, 5]. The health consequences of alcohol consumption on cardiovascular diseases are complex and controversial [6]. On the one hand, an estimated of 3.8% of all global deaths and 9.7% of global burden from cardiovascular diseases in men were attributed to alcohol consumption [7]. On the other hand, Fairbairn et al. found that male drinkers gained greater benefits from alcohol consumption than women, who tended to increase positive mood and were more likely to social contact [8]. Social contact was negatively related with mood disorders, whereas mood disorders were associated with increased risk of incident cardiovascular reactivity [9, 10]. And several epidemiologic studies of diverse population showed that moderate alcohol consumption was associated with a decreased risk of cardiovascular diseases incidence and/or mortality [11–15]. However, most of these studies focused on average ethanol consumption and they did not explore the effect of drinking pattern on CHD incidence. Daily or weekly alcohol consumption alone is not adequate to describe the relationship between alcohol consumption and incident CHD and drinking pattern may play an important role in the alcohol-CHD relationship [16]. In addition, there is limited evidence on association between drinking pattern and CHD incidence among the middle-aged and older population in China [17].

Alcohol consumption was a common practice, especially in China [18]. However, Chinese drinking cultural is quite different from that in western countries, and the main differences are described as follows: 1) the highest ethanol content of Chinese strong liquor is over 53% (v/v) while the spirit wine in western countries is less than 40% (v/v). In western countries, people usually drink liquor by mixing them with other juice or beverages, which may dilute actual ethanol content [19]; 2) Chinese drinkers tend to urge their friends to drink at a social drinking atmosphere and heavy drinking often occurs as a result of repetitive toasting [20], while solitary drinking in wine bar and party is relatively common in western drinkers [21]; 3) studies on alcohol use disorders in China exhibited a different demographic profile from that reported in western population [22, 23]. Alcohol use disorders are typically common among male drinkers and middle-aged individuals [24].

Therefore, it is necessary and valuable to investigate the association between drinking pattern and incident CHD among the middle-aged and older Chinese population.

## Methods

### Study population

Our study was based on the Dongfeng-Tongji (DFTJ) cohort, the design and methods of DFTJ cohort have been previously described elsewhere [25]. Briefly, a total of 27,009 retired employees of Dongfeng Motor Corporation were enrolled in the baseline survey between September 2008 and June 2010. All participants completed questionnaires and provided fasting blood samples for medical examination. Five years later, a total of 25,978 participants (the rate of follow-up was 96.2%) completed the first follow-up until October 2013.

In the present study, we excluded participants who had CHD, stroke or cancer at baseline (n = 5,865), those with missing information on alcohol consumption (n = 17) and other covariates (n = 843). Given the low percentages of female drinkers (6.51%), we further excluded women from our analysis and a total of 8,469 eligible male subjects were included in this analysis. The Medical Ethics Committee of School of Public Health, Tongji Medical College, Huazhong University of Science and Dongfeng Central Hospital approved the study protocols. All participants provided their written informed consent.

### Ascertainment of CHD

Incident CHD was defined as the first hospital admission with an occurrence of non-fatal myocardial infarction, stable and unstable angina pectoris, coronary artery bypass graft or percutaneous coronary intervention and CHD death [26]. The diagnosis of CHD was based on health-care service system of Dongfeng Motor Corporation and electronic medical records in the hospitals of Dongfeng Motor Corporation made by cardiologists. CHD death was confirmed on the basis of the underlying of death on death certificate according to ICD-10:I20-I25 [27, 28].

### Alcohol consumption and covariates

Information on alcohol consumption was collected by a semi-structured questionnaire (S1 File). Participants who were drinking alcoholic beverages at least one time per week for more than six months were defined as current drinkers and they were asked to answer the following questions regarding drinking frequency, type, average amount once a time, and age of drinking. Those who had abstained the previous drinking six months or more were defined as former drinkers. The ethanol consumption in grams per times was calculated as the sum of average ethanol content per type of alcoholic beverages multiplied by volumes of alcoholic beverages per times, the daily average ethanol consumption was multiplied by ethanol amounts consumption per times and drinking frequency. The average ethanol content (v/v) of liquor, beer, and wine was 42%, 4%, and 12%, respectively.

Information regarding demographic characteristics, socio-economic status, disease histories, lifestyle (smoking status, alcohol consumption, physical activity, et al.) and dietary habits was collected through face-to-face questionnaire interviews. The design of semi-structured questionnaire was based on literatures research, consultation of experts and pre-survey. The pre-survey had validated the feasibility and validity of the questionnaire. Participants who were smoking at least one cigarette per day over the past six months were defined as current smokers. Physical activity was defined as individuals who regularly exercise more than 20 min per time and more than three times per week over the last six months [29]. Standing weight, height and waist circumference were made at health examination center in Dongfeng General Hospital. Body mass index (BMI) was calculated by dividing weight in kilograms by height in meters squared [30]. Diseases were verified by self-reported physician diagnosis, medical examination, or current use of medication. Diabetes was defined as individuals with a previous self-reported physician diagnosis of diabetes, or fasting glucose level ≥ 7.0 mmol/L, or current use of oral hypoglycemic medication or insulin; Hypertension was defined as subjects with blood pressure ≥ 140/90 mmHg at medical examination, or taking antihypertensive medication, or a self-reported of physician diagnosis of hypertension; Hyperlipidemia was defined as participant with a previous physician diagnosis of hyperlipidemia, or current use of lip-lowering medication, or total cholesterol (TC) > 5.72 mmol/L or total triglycerides (TG) > 1.70 mmol/L at medical examination [25, 26].

Blood lipids, hepatic function, renal function and tumor-associated antigens were determined by the ARCHITECTC i8200 automatic analyzer (Abbott Park, Illinois, USA) and fasting glucose levels were measure via Aeroset automatic analyzer (Abbott Park, Illinois, USA) at the laboratory of Dongfeng Central Hospital.

### Statistical analysis

Continuous characteristics were presented as mean (standard deviation) and categorical variables were summarized as count (percentage). Differences in baseline characteristics by drinking status were evaluated using a Chi-square test for categorical variables and a one-way analysis of variance (ANOVA) for continuous variables. Cox proportional hazard model was conducted to estimate the adjusted hazard ratios (HRs) and 95% confidence intervals (CIs) of incident CHD in relation to alcohol consumption. We applied two multivariate hazard models, model 1 adjusted for age, education level and smoking status and model 2 additionally adjusted for BMI, family history of CHD, diabetes, hypertension, hyperlipidemia and physical activity. Stratified analyses were performed by mainly baseline characteristics [including current smoking (yes, no), BMI (< 25, ≥ 25 kg/m^2^), diabetes (yes, no), hypertension (yes, no), and hyperlipidemia (yes, no)]. All statistical analyses were performed using SPSS software version 20.0 and the *p*-value of less than 0.05 (two sides) was considered statistically significant.

## Results

After an average of 4.36 years of follow-up (36,953 person-years), we identified a total of 959 incident CHD cases. Baseline characteristics of the study participants were presented in Table 1. Of the 8,469 men aged 45–81 years, 4,096 (48.36%) participants were defined as non-drinkers and 3,616 (42.7%) participants were current drinkers. At baseline, the mean age of total participants was 65.43 (SD = 6.55) years, mean BMI was 24.37 (SD = 3.20) kg/m^2^. Current drinkers tended to be younger, more likely to be smokers, less educated and presented smaller percentages of diabetes, hypertension and hyperlipidemia. They were also more likely to be physical active, had higher level of AST, TC and HDL concentration and had lower level of ALT. No significant differences regarding BMI, family history of CHD, TG and LDL-C level were observed between current drinkers and non-drinkers.

**Table 1:** Baseline characteristics of study participants.

Variables	Non-drinkers	Current drinkers	Former drinkers	*P* value
Sample size	4096	3616	757	
Case, N (%)	500 (12.21)	375 (10.37)	84 (11.10)	0.04
Age (years)	66.32 (6.90)	64.37 (6.06)	65.68 (6.08)	<0.001
Education < High School, N (%)	2436 (59.47)	2441 (67.50)	507 (66.97)	<0.001
Current smoker, N (%)	1142 (27.88)	1960 (54.20)	266 (35.14)	<0.001
BMI (kg/m^2^)	24.39 (3.24)	24.30 (3.13)	24.60 (3.23)	0.06
Physical activity (hours/wk)	8.16 (9.05)	9.26 (14.70)	9.69 (13.39)	<0.001
TG (mmol/L)	1.38 (0.99)	1.43 (1.31)	1.36 (0.88)	0.13
TC (mmol/L)	4.97 (0.92)	5.08 (0.91)	4.95 (0.87)	<0.001
HDL-C (mmol/L)	1.35 (0.38)	1.44 (0.39)	1.35 (0.36)	<0.001
LDL-C (mmol/L)	2.95 (0.78)	2.95 (0.78)	2.92 (0.73)	0.64
Fasting glucose (mmol/L)	6.15 (1.83)	6.01 (1.59)	6.45 (2.20)	<0.001
AST (mmol/L)	25.98 (18.37)	27.78 (18.69)	26.29 (15.37)	<0.001
ALT (mmol/L)	26.32 (29.96)	25.00 (17.53)	26.76 (23.60)	0.05
Diabetes, N (%)	795 (19.4)	527 (14.6)	191 (25.2)	<0.001
Hypertension, N (%)	1364 (33.3)	1129 (31.2)	306 (40.4)	<0.001
Hyperlipidemia, N (%)	985 (24.0)	782 (21.6)	246 (32.5)	<0.001
Family history of CHD, N (%)	96 (2.34)	97 (2.68)	12 (1.59)	0.17

Continuous variables were presented as mean (SD); categorical variables were summarized as count (percentage). BMI, body mass index; TG, total triglyceride; TC, total cholesterol; HDL-C, high-density lipoprotein cholesterol; LDL-C, low-density lipoprotein cholesterol; AST, aspartate aminotransferase; ALT, alanine aminotransferase

Adjusted HRs (95% CIs) for incident CHD in relation to alcohol consumption were presented in Table 2. After adjusting for age, education, smoking status, BMI, family history of CHD, diabetes, hypertension, hyperlipidemia and physical activity, current drinkers were associated with a 16% lower risk of CHD incidence compared with non-drinkers (HR = 0.84, 95% CI = 0.73, 0.98).

**Table 2:** Adjusted HR (95% CI) for incident CHD according to alcohol consumption.

Variables	Non-drinkers	Current drinkers	Former drinkers
Sample size	4096	3616	757
Cases/Person-years	500/17862	375/15830	84/3261
Model 1	Reference	0.86 (0.74, 0.99)	0.98 (0.78, 1.24)
Model 2	Reference	0.84 (0.73, 0.98)	0.84 (0.85, 1.07)

Model 1: adjusted for age, education level and smoking status. Model 2: additionally adjusted for BMI, family history of CHD, diabetes, hypertension, hyperlipidemia and physical activity.

Then we further explored the association of drinking pattern with incident CHD. Table 3 showed the adjusted HRs (95% CIs) for incident CHD in relation to the ethanol amounts once a time. Participants who consumed 20.1–40 gram ethanol once a time were associated with a decreased risk of CHD incidence (HR = 0.76, 95% CI = 0.62, 0.94) in model 2. Moderate ethanol consumption once a time was associated with a reduced risk of CHD incidence among current smokers (HR = 0.73, 95% CI = 0.54, 0.99) and individuals with hypertension (HR = 0.67, 95% CI = 0.49, 0.92).

**Table 3:** Adjusted HRs (95% CIs) for incident CHD according to the ethanol amounts once a time.

Variables	Non-drinkers	0.01–20 g/time	20.01–40 g/time	> 40 g/time	*P* value^a^
**Total**					
Sample size	4096	1111	1311	1149	
Cases/Person-years	500/17862	129/4847	127/5756	115/4793	
Model 1	Reference	0.99 (0.82, 1.21)	0.76 (0.62, 0.93)	0.84 (0.67, 1.03)	
Model 2	Reference	1.01 (0.83, 1.24)	0.76 (0.62, 0.94)	0.86 (0.69, 1.06)	
**Current Smoking**					0.64
Yes (n = 3,080)	Reference	1.08 (0.81, 1.45)	0.73 (0.54, 0.99)	0.88 (0.65, 1.17)	
No (n = 4,587)	Reference	0.94 (0.71, 1.24)	0.83 (0.62, 1.11)	0.84 (0.60, 1.17)	
**Body Mass Index**					0.19
≥ 25.00 (n = 3,147)	Reference	0.92 (0.68, 1.25)	0.82 (0.61, 1.10)	0.81 (0.58, 1.12)	
< 25.00 (n = 4,520)	Reference	1.17 (0.90, 1.52)	0.80 (0.60, 1.07)	1.00 (0.76, 1.33)	
**Diabetes Mellitus**					0.13
Yes (n = 1,319)	Reference	1.20 (0.79, 1.82)	0.72 (0.44, 1.71)	0.75 (0.46, 1.22)	
No (n = 6,348)	Reference	1.00 (0.80, 1.26)	0.82 (0.65, 1.03)	0.95 (0.75, 1.20)	
**Hypertension**					0.29
Yes (n = 2,482)	Reference	0.74 (0.54, 1.01)	0.67 (0.49, 0.92)	0.71 (0.51, 1.00)	
No (n = 5,185)	Reference	1.31 (1.01, 1.69)	0.87 (0.67, 1.14)	1.04 (0.79, 1.37)	
**Hyperlipidemia**					0.56
Yes (n = 1,761)	Reference	0.83 (0.56, 1.22)	0.80 (0.54, 1.18)	0.76 (0.51, 1.11)	
No (n = 5,906)	Reference	1.13 (0.90, 1.42)	0.79 (0.62, 1.01)	0.94 (0.73, 1.21)	

Model 1: adjusted for age, education level and smoking status. Model 2: additionally adjusted for BMI, family history of CHD, diabetes, hypertension, hyperlipidemia and physical activity.

^a^*P* value for the interaction term of continuous biomarker*categorical stratifying variable.

We observed no statistically significant association between type and frequency of alcohol consumption and CHD incidence among total participants (presented in Table 4 and S1 Table). The HRs (95% CIs) for incident CHD in subjects who consumed liquor, beer, wine, and mixed alcoholic beverages were 0.85 (0.71–1.02), 1.00 (0.72–1.40), 0.81 (0.43–1.51), and 0.88 (0.72–1.07), respectively. In the stratified analyses, we found that the association between drinking frequency and risk of CHD incidence was more prominent among subject with diabetes, hypertension, or hyperlipidemia. A significant interaction between drinking frequency and diabetes on the risk of incident CHD was also observed (*P*_interaction_ = 0.03).

**Table 4:** Adjusted HRs (95% CIs) for incident CHD according to type of alcohol consumption.

Variables	Non-drinkers	liquor	beer	red wine	Mixed^b^
Sample size	4096	1745	353	92	1401
Cases/Person-years	500/17862	181/7686	353/526	11/406	144/6141
Model 1	Reference	0.84 (0.70–1.00)	1.00 (0.72–1.38)	0.87 (0.48–1.59)	0.85 (0.71–1.04)
Model 2	Reference	0.85 (0.71–1.02)	1.00 (0.72–1.40)	0.81 (0.43–1.51)	0.88 (0.72–1.07)

^b^the type of alcoholic beverages is greater than or equal two.

Model 1: adjusted for age, education level and smoking status. Model 2: additionally adjusted for BMI, family history of CHD, diabetes, hypertension, hyperlipidemia and physical activity.

Table 5 showed the association between daily ethanol consumption and incident CHD. A statistically significant inverse association was observed between daily alcohol consumption and CHD incidence, HRs (95% CIs) for incident CHD in the first, second, and third daily ethanol consumption were 0.80 (0.65–0.99), 1.02 (0.84–1.22), and 0.75 (0.59–0.96), respectively, compared with non-drinkers. In stratified analyses, the relationships were generally consistent across the categories. There was an interaction effect between the daily ethanol consumption and diabetes mellitus on the risk of incident CHD (*P*_interaction_ = 0.01).

**Table 5:** Adjusted HRs (95% CIs) for incident CHD according to daily ethanol consumption.

Variables	Non-drinkers	0.01–10 g/day	10.01–30 g/day	> 30 g/day	*P* value^a^
**Total**					
Sample size	4096	1101	1448	995	
Cases/Person-years	500/17862	110/4865	172/6289	86/4385	
Model 1	Reference	0.78 (0.63, 0.96)	1.03 (0.86, 1.23)	0.71 (0.56, 0.90)	
Model 2	Reference	0.80 (0.65, 0.99)	1.02 (0.84, 1.22)	0.75 (0.59, 096)	
**Current Smoking**					0.99
Yes (n = 3,062)	Reference	0.87 (0.64, 1.19)	1.02 (0.78, 1.34)	0.72 (0.52, 1.01)	
No (n = 4,578)	Reference	0.76 (0.57, 1.03)	1.01 (0.78, 1.31)	0.83 (0.57, 1.20)	
**Body Mass Index**					0.22
≥ 25.00 (n = 3,135)	Reference	0.82 (0.60, 1.13)	0.92 (0.70, 1.22)	0.80 (0.56, 1.14)	
< 25.00 (n = 4,505)	Reference	0.86 (0.65, 1.15)	1.21 (0.95, 1.54)	0.81 (0.59, 1.12)	
**Diabetes Mellitus**					0.01
Yes (n = 1,314)	Reference	1.14 (0.77, 1.70)	0.89 (0.58, 1.37)	0.48 (0.24, 0.96)	
No (n = 6,326)	Reference	0.74 (0.58, 0.95)	1.10 (0.90, 1.35)	0.86 (0.66, 1.11)	
**Hypertension**					0.29
Yes (n = 2,475)	Reference	0.70 (0.51, 0.96)	0.76 (0.56, 1.01)	0.65 (0.44, 0.95)	
No (n = 5,165)	Reference	0.91 (0.68, 1.21)	1.32 (1.04, 1.67)	0.87 (0.63, 1.19)	
**Hyperlipidemia**					0.76
Yes (n = 1,757)	Reference	0.83 (0.57, 1.20)	0.88 (0.62, 1.26)	0.62 (0.39, 1.00)	
No (n = 5,883)	Reference	0.82 (0.64, 1.06)	1.12 (0.90, 1.38)	0.82 (0.62, 1.09)	

Model 1: adjusted for age, education level and smoking status. Model 2: additionally adjusted for BMI, family history of CHD, diabetes, hypertension, hyperlipidemia and physical activity.

^a^*P* value for the interaction term of continuous biomarker*categorical stratifying variable.

Additionally, we took the ethanol amounts once a time and frequency into comprehensive consideration by dividing current drinkers into different subgroups according to their drinking pattern. Table 6 presented HRs and 95% CI for incident CHD across all degree of ethanol amounts once a time according to drinking frequency in comparison with non-drinkers. Compared with non-drinkers, participants who consumed alcoholic beverages less than 5 times/week and 20.01–40 /time had a decreased risk of CHD incidence (HR = 0.73, 95% CI = 0.52, 0.96).

**Table 6:** HRs (95% CIs) across all degrees of ethanol amounts once alcohol consumption according to drinking frequency.

		0.01–20 g/time	20.01–40 g/time	>40 g/time
1–4 times/week	Participants	640	637	535
	Model 2	0.911 (0.70, 1.19)	0.73 (0.52, 0.96)	1.05 (0.80, 1.38)
≥ 5 times/week	Participants	463	661	556
	Model 2	1.17 (0.90, 1.53)	0.81 (0.61, 1.08)	0.73 (0.54, 1.00)

The reference subjects were the participants who were defined as non-drinkers.

Model 2: adjusted for age, education level, smoking status, BMI, family history of CHD, diabetes, hypertension, hyperlipidemia and physical activity.

## Discussion

This prospective cohort study revealed an inverse association between alcohol consumption and incident CHD among middle-aged and older Chinese adults. With respect to the features of drinking, moderate quantity of ethanol amounts once a time and daily ethanol consumption were associated with a lower risk of CHD incidence Participants who consumed moderate quantity of ethanol amounts once a time with lower drinking frequency had the lowest risk of incident CHD, which indicated that drinking patterns might play an important protective role in the relationship between alcohol consumption and CHD incidence.

Our results were consistent with previous studies on the associations between alcohol consumption and cardiovascular diseases incidence and/or mortality. A meta-analysis of 84 articles revealed that moderate alcohol consumption was associated with a 25–35% lower risk of multiple cardiovascular outcomes and that protective effect was also manifested with heavy drinkers [31]. Mukamal et al. examined the associations between drinking pattern and risk of myocardial infarction (MI) incidence among 38,077 male, they found a grade and inverse association between average alcohol consumption and risk of incident MI and drinking pattern played an important protective role in MI incidence [32]. A cohort study of Spanish population (European Prospective Investigation into Cancer study, EPIC) showed that alcohol consumption was associated with a 30% lower risk of CHD among men aged 29–69 years [13]. To the contrary, Corrao et al. found that regular moderate alcohol consumption was associated with a reduced risk of CHD incidence whereas heavy drinking was related to a higher risk of CHD incidence [33]. An updated meta-analysis of 34 prospective studies suggested a J-shaped relationship between alcohol consumption and total mortality in both men and women [34]. However, former drinkers who stopped drinking owning to health problem were included as reference group in this meta-analysis.

Bazzano et al. found that average alcohol consumption was related to lower incidence and mortality of MI and CHD among 64,597 middle-aged and older Chinese men across 15 provinces in mainland China [35]. But they did not separate the former drinkers from reference group, which might contribute to a misleadingly elevated risk as most former drinkers had stopped drinking for illness. In addition, there are tremendous differences in the drinking habit and CHD incidence between northern and southern China and they did not evaluate the relationships between the drinking pattern and risk of CHD incidence.

In our analyses, the variable of daily ethanol consumption was calculated as ethanol amounts once a time multiplied by drinking frequency, when we classified the current drinkers into different subgroups according to their daily ethanol consumption, participants who had different drinking pattern would classify into the same subgruops and misclassification bias would affect the relationship between alcohol consumption and incident CHD. The accurate balance between the rapid oxidation of ethanol and acetaldehyde is the key element of acetaldehyde concentrations within cells [36]. Different drinking pattern could affect this delicate balance, which led toxic and oxidize damage in one or more tissues [37]. Therefore, average alcohol consumption alone was not adequate to evaluate the relationship between alcohol consumption and incident CHD, ethanol amounts once a time and drinking frequency played an important role in the mechanisms underlying that relationship. We independently analyzed the effect of the ethanol amounts once a time or drinking frequency on incident CHD and took those two important features of drinking into comprehensive consideration. Our study suggested that moderate quantity of ethanol amounts once a time with lower drinking frequency was a positive drinking pattern for CHD incidence among the middle-aged and older men.

Our study has several strengths. Firstly, the questionnaires provided us with detailed information on alcohol consumption and we took the ethanol amounts once a time and drinking frequency into comprehensive consideration. Secondly, we excluded the former drinkers from the reference group which might minimize bias to some extent. In addition, all participants were covered by health-care service system of Dongfeng Motor Corporation and each participant had a unique medical insurance ID, providing easy access to personal information and medical records.

Some limitations of our study should also be addressed. First, the information on alcohol consumption and other covariates based on self-reported and the amounts of alcohol consumption might be underestimated. Second, participants included in this study were middle-aged and older Chinese men without CHD, stroke, and cancer at baseline, thus further studies are needed to validate the association between drinking pattern and incident CHD among the general population of different ages or health status. Although we controlled a variety of established confounders that could affect the association between drinking pattern and incident CHD in multivariate mode, the potential residual confounding factors could not be eliminated in observational studies.

In conclusion, alcohol consumption was associated with a decreased risk of CHD incidence in middle-aged and older Chinese men. Moderate quantities of ethanol amounts once a time with lower drinking frequency could be considered as a favorable drinking pattern for the middle-age and older men. Because of the features of drinking are modifiable, changes of drinking pattern may be beneficial to CHD control and prevention. However, individual difference and potential cardiovascular benefits should been taken into consideration on the recommendation of drinking and any strategy regarding alcohol consumption should synthesize its potential harm as well as benefits. Our findings provide a novel standpoint for estimating the health influence of drinking. Advice regarding alcohol consumption should not be only based on the drinking amounts, but also focus on drinking pattern.

## Supporting information

S1 File Figure A. semi-structured questionnaire regarding alcohol consumption in English; Figure B. semi-structured questionnaire regarding alcohol consumption in original language (Chinese).
(DOCX)

S1 Table Adjusted HRs (95% CIs) for incident CHD according to the drinking frequency. (DOCX)
