# Supplementary material for: Association of drinking pattern with risk of coronary heart disease incidence in the middle-aged and older Chinese men: Results from the Dongfeng-Tongji cohort
Source: PLoS One. 2017 May 25;12(5):e0178070. doi: 10.1371/journal.pone.0178070 (PMC5444775; doi:10.1371/journal.pone.0178070)
Supplement: S1 File — Figure A. semi-structured questionnaire regarding alcohol consumption in English; Figure B. semi-structured questionnaire regarding alcohol consumption in original language (Chinese). (DOCX) [file pone.0178070.s001.docx]

**Supporting information**

Questionnaire on alcohol consumption

| E02a alcohol consumption | □ current drinking | E02b age of starting drinking, □□years | |
| --- | --- | --- | --- |
|  |  | E02c drinking frequency (times/week) □≤1 □2-4 □5-7 □8-10 □ ＞10 | |
|  |  |  |  |
|  |  | E02d drinking type and average amount once a time (multiple selection) | □liquor, □□.□ liang (50 ml) |
|  |  |  | □bear, □□.□ bottle (500 ml) |
|  |  |  | □wine, □□.□ liang (50 ml) |
|  | □ former drinking | E02e age of starting drinking, □□years | |
|  |  | E02f former drinking frequency (times/week) □≤1 □2-4 □5-7 □8-10 □ ＞10 | |
|  |  |  |  |
|  |  | E02h former drinking type and average amount once a time (multiple selection) | □liquor, □□.□ liang (50 ml) |
|  |  |  | □bear, □□.□ bottle (500 ml) |
|  |  |  | □wine, □□.□ liang (50 ml) |
|  |  | E02g age of quitting drinking, □□years | |
|  | □ never drinking or occasional drinking (less than 1 times/week) | | |

Fig A. Semi-structured questionnaire regarding alcohol consumption in English


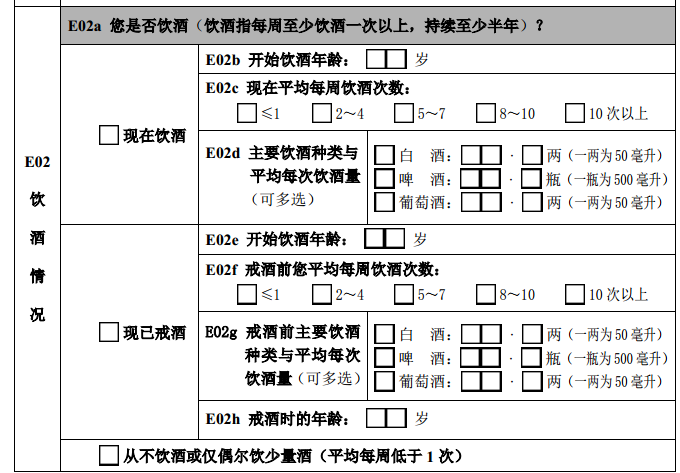


Fig B. Semi-structured questionnaire regarding alcohol consumption in original language (Chinese)
