# Supplementary material for: Association of drinking pattern with risk of coronary heart disease incidence in the middle-aged and older Chinese men: Results from the Dongfeng-Tongji cohort
Source: PLoS One. 2017 May 25;12(5):e0178070. doi: 10.1371/journal.pone.0178070 (PMC5444775; doi:10.1371/journal.pone.0178070)
Supplement: S1 Table — (DOCX) [file pone.0178070.s002.docx]

**S1 Table Adjusted HRs (95% CIs) for incident CHD according to the drinking frequency**

| **Variables** | **Non-drinkers** | **1 time/week** | **2-4 times/week** | **5-7 times/week** | **>7 times/week** | ***p* value^a^** |  |
| --- | --- | --- | --- | --- | --- | --- | --- |
| **Total** |  |  |  |  |  |  |  |
| Sample size | 4096 | 695 | 1173 | 958 | 762 |  |  |
| Cases/Person-years | 500/17862 | 76/3057 | 119/5153 | 95/4188 | 82/3307 |  |  |
| Model 1 | Reference | 0.80 (0.63, 1.03) | 0.91 (0.74, 1.12) | 0.83 (0.66, 1.04) | 0.88 (0.69, 1.12) |  |  |
| Model 2 | Reference | 0.84 (0.66, 1.08) | 0.95 (0.77, 1.17) | 0.91 (0.72, 1.14) | 0.96 (0.75, 1.23) |  |  |
| **Current Smoking** |  |  |  |  |  | 0.45 |  |
| Yes (n=3,084) | Reference | 0.80 (0.55, 1.16) | 0.92 (0.68, 1.25) | 0.97 (0.71, 1.34) | 0.80 (0.57, 1.31) |  |  |
| No (n=4,600) | Reference | 0.84 (0.60, 1.18) | 0.90 (0.67, 1.21) | 0.74 (0.52, 1.05) | 1.12 (0.78, 1.61) |  |  |
| **Body Mass Index** |  |  |  |  |  | 0.39 |  |
| ≥ 25.00 (n=3,147) | Reference | 0.84 (0.59, 1.20) | 0.79 (0.58, 1.09) | 1.03 (0.74, 1.43) | 0.79 (0.53, 1.18) |  |  |
| < 25.00 (n=4,537) | Reference | 0.86 (0.61, 1.22) | 1.09 (0.83, 1.44) | 0.83 (0.60, 1.14) | 1.10 (0.80, 1.50) |  |  |
| **Diabetes Mellitus** |  |  |  |  |  | 0.03 |  |
| Yes (n=1,317) | Reference | 0.81 (0.70, 1.14) | 0.75 (0.54, 1.03) | 0.71 (0.49, 1.02) | 0.51 (0.38, 0.89) |  |  |
| No (n=6,367) | Reference | 0.73 (0.54, 0.99) | 0.95 (0.75, 1.20) | 0.92 (0.71, 1.81) | 1.03 (0.79, 1.34) |  |  |
| **Hypertension** |  |  |  |  |  | 0.15 |  |
| Yes (n=2,486) | Reference | 0.81 (0.57, 1.15) | 0.74 (0.54, 1.03) | 0.70 (0.48, 1.01) | 0.58 (0.38, 0.88) |  |  |
| No (n=5,198) | Reference | 0.81 (0.70, 1.16) | 1.09 (0.83, 1.43) | 1.05 (0.78, 1.40) | 1.22 (0.90, 1.66) |  |  |
| **Hyperlipidemia** |  |  |  |  |  | 0.57 |  |
| Yes (n=1,763) | Reference | 0.88 (0.58, 1.32) | 0.84 (0.57, 1.24) | 0.89 (0.57, 1.39) | 0.53 (0.31, 0.93) |  |  |
| No (n=5,921) | Reference | 0.80 (0.59, 1.10) | 0.97 (0.75, 1.24) | 0.90 (0.70, 1.17) | 1.06 (0.81, 1.40) |  |  |

Model 1: adjusted for age, education level and smoking status.

Model 2: additionally adjusted for BMI, family history of CHD, diabetes, hypertension, hyperlipidemia and physical activity.

^a^P value for the interaction term of continuous biomarker*categorical stratifying variable.
